# Rapid fine mapping of causative mutations from sets of unordered, contig-sized fragments of genome sequence

**DOI:** 10.1186/s12859-018-2515-5

**Published:** 2019-01-07

**Authors:** Ghanasyam Rallapalli, Pilar Corredor-Moreno, Edward Chalstrey, Martin Page, Daniel MacLean

**Affiliations:** 1 The Sainsbury Laboratory, Norwich Research Park, Colney Lane, Norwich, NR4 7UH UK; 2 Norwich Medical School, University of East Anglia, James Watson Road, Norwich, NR4 7UQ UK; 3 John Innes Centre, Norwich Research Park, Colney Lane, Norwich, NR4 7UH UK; 4 Birkbeck, University of London, Bloomsbury, London, WC1E 7HX UK

**Keywords:** Mapping by sequencing, Next generation mapping, Bulk segregant analysis

## Abstract

**Background:**

Traditional Map based Cloning approaches, used for the identification of desirable alleles, are extremely labour intensive and years can elapse between the mutagenesis and the detection of the polymorphism. High throughput sequencing based Mapping-by-sequencing approach requires an ordered genome assembly and cannot be used with fragmented, un-scaffolded draft genomes, limiting its application to model species and precluding many important organisms.

**Results:**

We addressed this gap in resource and presented a computational method and software implementations called CHERIPIC (Computing Homozygosity Enriched Regions In genomes to Prioritise Identification of Candidate variants). We have successfully validated implementation of CHERIPIC using three different types of bulk segregant sequence data from Arabidopsis, maize and barley, respectively.

**Conclusions:**

CHERIPIC allows users to rapidly analyse bulk segregant sequence data and we have made it available as a pre-packaged binary with all dependencies for Linux and MacOS and as Galaxy tool.

**Electronic supplementary material:**

The online version of this article (10.1186/s12859-018-2515-5) contains supplementary material, which is available to authorized users.

## Background

Forward genetic screens are an essential and widely used tool for the identification of alleles underlying desirable traits [1]. The successful identification and cloning of genes from these screens depends on availability of polymorphic molecular markers between two accessions and a physical map of markers [2]. A mapping population is created by crossing a mutated plant from one polymorphic accession with another. Map based Cloning (MBC) involves screening of either individuals or pooled individuals from segregating populations using defined markers to identify regions of the genome with limited or no recombination due to linkage disequilibrium and refine the mutant position [3–6]. Traditional MBC is labour intensive and years can elapse between the mutagenesis and the detection of the polymorphism responsible [2]. Mapping-by-sequencing (MBS) is a high throughput sequencing based mutation mapping approach that has shortened this process considerably by allowing the calculation of allele frequency from bulks and the identification of causal mutations at single-nucleotide resolution [7]. However, application of MBS requires a chromosomal ordered genome assembly and cannot be used with fragmented, unscaffolded draft genomes, thus limiting its application to model species and precluding many important organisms. Most crops and their wild relatives have complex and difficult to order genomes, hence genetic and genomic resources remain in draft stages. Carrying out mapping studies without the necessary genetic resources is cumbersome, thus limiting the number of mapping studies in non-model organisms and rendering a store of potential disease resistances and other agronomically important traits unavailable.

We present a computational method and software implementations to address this problem, called CHERIPIC (Computing Homozygosity Enriched Regions In genomes to Prioritise Identification of Candidate variants). CHERIPIC makes use of short contig fragments (such as those from the first pass assembly of Illumina data or a PacBio sequence run) from bulk segregant sequencing (BSS) experiments to call variants and to reduce the list of candidates to a few closely linked variants in the region harbouring the trait of interest and in some cases includes the candidate mutation as well. We successfully applied CHERIPIC to fragmented assemblies made from publicly available BSS high-throughput data for Arabidopsis, maize and barley. CHERIPIC improves on previous methods by being input type agnostic, working well on genome-seq and RNA-seq data, having extremely low computational requirements and being available for direct use through a web interface.

## Implementation

**Fig. 1:**
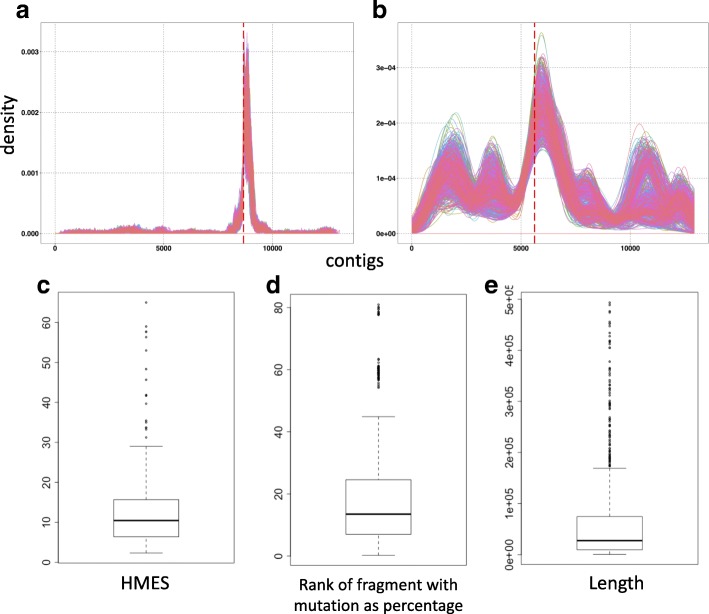
Region of the genome harbouring the candidate mutation is defined by the assembled fragments with enriched homozygosity. (a,b) Density plots of fragments *HMES* from 1000 iterations for an (**a**) outcross data and (**b**) backcross data are plotted along the entire Arabidopsis genome. Contigs from chromosome 1 to 5 are arranged sequentially. Causative mutation location is highlighted using a red vertical dashed line. (c,d,e) Boxplots showing the distribution of (**c**) *HMES* (**d**) *HMES* rank presented as a percentage of all fragments with *HMES* >1 and (**e**) length of the fragment with candidate mutation from 1000 iterations

The process of mutation and crossing results in reduction of the density of experimentally induced homozygous polymorphisms relative to heterozygous polymorphisms as distance from the causative mutation increases [7, 8]. We assessed the properties of fragmented genome assemblies of Arabidopsis with respect to statistics of allele frequency. The length distribution of scaffolds was modelled and followed a log-normal distribution (Additional file 1: Figure S1, *R*^2^=0.796). Therefore we generated 1000 simulated Arabidopsis assemblies containing fragments of length fitting a log-normal distribution for both backcross and outcross bulk segregant sequence data. Variants were called on Arabidopsis genome for both mapping experiments. These variants (as VCF files) were used in the simulations to generate both fragmented assemblies and associated variant calls from both mapping populations (see methods for further details). We defined a straightforward homozygosity enrichment score (*HMES*) strictly the ratio of homozygous to heterozygous variants. *HMES*=(*α*+*ρ*)/(*β*+*ρ*), where *α* = number of homozygous variants on the selected fragment, *β* = number of heterozygous variants on the selected fragment and *ρ* = a ratio adjustment factor, included to avoid division of or by zero and *ρ* = 0.5 is used in our case. For each fragment, *HMES* serves as an estimator of the nearness of the fragment to the causative mutation. The density of fragment *HMES* is plotted against a continuous genome in Fig. 1 for each of the thousand simulations. The outcross (Fig. 1a) and backcross (Fig. 1b) data both show strong enrichment of *HMES* around the causative mutation, though backcross data show strong peaks elsewhere, a consequence of lower density of SNPs resulting from mutagenesis. In our outcross simulations, the fragment carrying the causative mutation has a mean *HMES* of 12.24 (median 10.45) and on average is in the top 18.8% (median 13.5%) of *HMES* scores (Fig. 1c, d). In our simulation length of the fragment ranged from 0.5kb to 493kb with median of 27.5kb. Together these statistics indicate that the *HMES* is a reliable way to identify the most likely fragments around or bearing the causative mutation.

We developed a rapid *HMES* sorting algorithm to arrange unordered fragments into a sequence representing distance from the causative mutation, though not necessarily their original order in the genome (Algorithm 1). In broad terms the algorithm orders the fragments with the largest *HMES* at the centre, the second largest to its left, the third largest to its right, the fourth largest to the extreme left and so on resulting in a rough ordering of the genomic fragments such that nearness to the centre of the ordering increases the likelihood of a fragment carrying the causative mutation (Additional file 1: Figure S2).


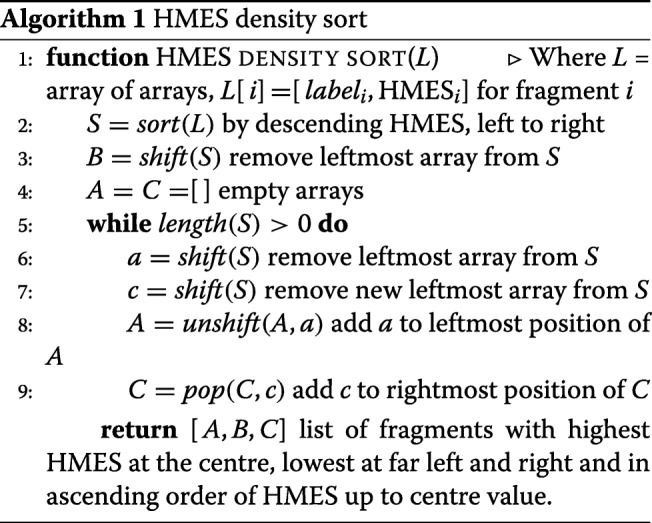


Output from CHERIPIC is a tab-delimited text file with the following information about the variants selected - “HMES, allele frequency, length of contig, id of contig, variant position in contig, reference base, coverage, read bases, base qualities, left sequence to variant, variant allele, right sequence to variant”. Left and right sequences are provided to easily design markers and sequence length can be user adjusted to retrieve enough sequence information.

## Results

We applied our algorithm to three plant genome data sets from previously published experiments in which sequence data are publicly available. Datasets being whole genome shotgun data of pooled bulks of the *sup2* Arabidopsis mutant (a mutation in *AT4G11260* at chromosome 4:6852405) [9], pooled bulk RNA-seq data from the maize *gl3* mutant (*GRMZM2G162434* gene at Chr4:185827677-185831259) [10] and exome capture data from the barley *mnd* mutant (*MLOC_64838* gene at Chr5:468277462-468279844) [11]. All data are from bulked segregant analysis involving outcrosses. In all these experiments, the allele under question is known to be recessive, hence we focussed on tracking the linkage disequilibrium around the mutant allele. For a recessive candidate in mutant bulks we expect an allele frequency close to 100%, while the allele frequency in background bulk would be around 33.3% (since two third fraction of the background are heterozygotes and half of that represent mutant allele), these percentages allow tuning of the identification of polymorphisms as homo/heterozygous according to calculated allele frequency. In all these data, we could show that *HMES* ordering can be used to narrow down the region of the genome to a fine interval to identify the causative mutation.

**Fig. 2:**
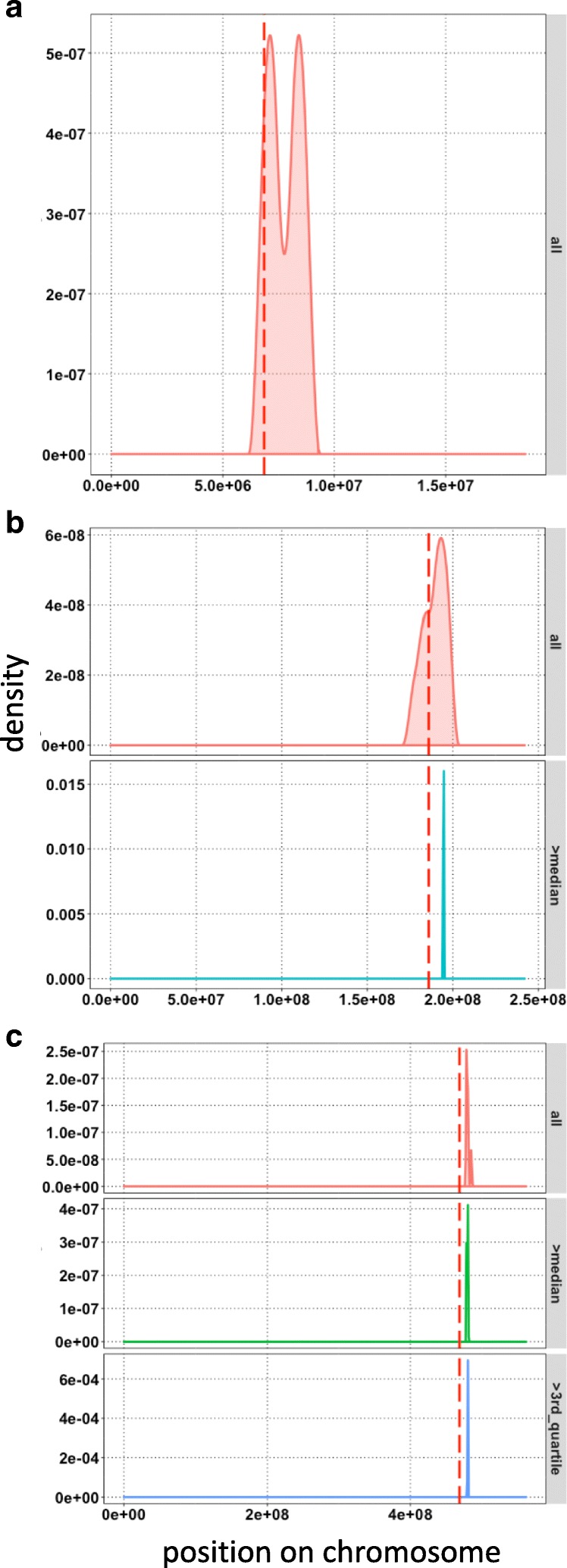
Implementation of CHERIPIC with bulk segregant sequencing data from plant species with varying genome sizes has identified variants very close to causative mutation. Density plots showing the position of selected variants (**a**) on Chromosome 4 of Arabidopsis for *sup2*; (**b**) on Chromosome 4 of maize for *gl3* and (**c**) on Chromosome 5 for *mnd* mutant as vertical red dotted lines. Top five percentile *HMES* variants were depicted for Arabidopsis and barley, while for maize all selected variants were depicted. In each panel, variant densities are presented as either all variants or variants with *HMES* grater than median or *HMES* grater than 3rd quartile. Variants with *HMES* from top 1% are very closely linked to causative mutation. Bulk segregant sequence data is from whole genome sequencing (Arabidopsis), RNA-seq (maize) and exome sequencing (barley) experiments

For the *sup2* Arabidopsis experimental data [9] we have generated an assembly from background bulks of *hasty* mutant data used in earlier simulations and used as a reference sequence for CHERIPIC and bulk variant calls. In addition, variant calls were made using both background parent and polymorphic parent (Col-T and Ws). CHERIPIC has identified 346 variants from fragments with *HMES*>1 (Additional file 1: Figure S3a). The mutation causing the *sup2* phenotype is on chromosome 4 and 97.68% (338 out of 346) of variants selected were on that chromosome (Additional file 1: Figure S3a). The variants from fragments most closely associated around causative mutation have a very high *HMES* (Fig. 1); therefore, we selected variants falling in top 5% of the score, resulting in 16 candidates (Fig. 2a and Additional file 1: Figure S3b). The closest variant is at a distance of 260 Kbp (260581bp) from the causative mutation reducing the area of the genome hosting the mutation to a very fine region. The candidate mutation causing the phenotype is not identified in our study, indicating the region of interest can still be found even in the absence of the causative mutation a strong advantage in noisy or false-positive/negative error prone SNP call sets. In this experiment, the bulks were sequenced at 8X coverage possibly resulting in non-detection of some of the variants, including the candidate mutation. The variants on selected fragments from CHERIPIC can be used as markers for further screening of bulks for segregation.

Bulk segregant data for the maize *gl3* [10] was generated using RNA-seq of pooled RNAs of the bulks. We first assembled transcripts from the RNA-seq data of non-*gl3* phenotype bulks and used this as the alignment reference sequence in CHERIPIC analysis. CHERIPIC identified twelve variants with *HMES* greater than one from all ten chromosomes (Additional file 1: Figure S4), ten of which on chromosome four (Fig. 2b), and collocated with the causative mutation. The distance between the closest and the causative mutation was 1Mbp (1006746 bp). A limitation of bulked RNA-seq approach is that polymorphisms may cause expression changes and transcripts from the gene carrying the causative mutation could be undetectable because of poor sequence coverage and could not be considered by CHERIPIC. Transcripts from *gl3* were at very low amounts and therefore missed.

Sequence data from barley *mnd* bulks was generated using exome capture [11]. Application of CHERIPIC to barley data has resulted in identification of 1997 variants with *HMES*>1 from all 7 Chromosomes (Additional file 1: Figure S5a). The causative mutation for *mnd* is located on chromosome 5 and 63.99% of selected variants (1278 out of 1997) were present on that chromosome (Additional file 1: Figure S5a). Selecting variants with top 5% of *HMES* resulted in 90 variants from all 7 Chromosomes (Additional file 1: Figure S5b), while 78 (86.67%) were on Chromosome 5 (Fig. 2c). Distance between *mnd* causative mutation and closest CHERIPIC variant was 11.7Mbp (11722011 bp). The barley *mnd* mutant was generated using X-ray mutagenesis which is known to create large deletions and could have added to the increased distance between the causative mutation and the closest variant identified in this case.

## Conclusions

To permit the easy application of our method and to allow users to rapidly analyse bulk segregant sequence data we have produced a range of implementations of the CHERIPIC algorithm. As input CHERIPIC takes a file of reference fragments and the variant files for both bulks. Variant calls from background bulks are not required, but if available help reduce the background and increase high quality variants for downstream analysis. Variants files can be provided as either pileup, BAM or VCF files. CHERIPIC is implemented in Ruby and is available as a pre-packaged binary with all dependencies for Linux and MacOS at https://github.com/TeamMacLean/cheripic. A Galaxy install script is provided to allow integration into to Galaxy servers. An interactive web interface is provided at http://cheripic.tsl.ac.uk.

## Methods

### Datasets

(1) Mapping population of a Arabidopsis leaf curl mutant (*hasty*) was generated through backcrossing it to the parent *mir159a*, a T-DNA insertion mutant [12]. From the mapping population, a bulk of 110 individuals showing mutant phenotype and a parent *mir159a* individual were sequenced to 50x coverage, using Illumina Hiseq2000 through paired end sequencing (2x100bp). (2) Two suppressor mutants (*sup#1* and *sup#2*) of hemizygous *uni-1D* transgene in Arabidopsis Ws background was out-crossed to Col-T, to generate mapping population [9]. Sequencing was done on (i) a pool of 80 individuals showing suppression phenotype, (ii) wild type Col-T and (iii) wild type Ws, using Illumina Genome Analyzer IIx as 75bp single end reads. We have used sequencing data from *sup#2*, Col-T and Ws in our analysis and these samples had 8.3 to 9.1x sequence coverage. (3) A maize mapping population was generated by out-crossing glossy phenotype showing *gl3-ref* allele in non-B73 genetic background to B73, an inbred reference line [10]. Sequencing was carried out on pools of RNA from 32 mutant phenotype individuals and 31 non-mutant phenotype individuals, on Illumina Genome Analyzer II as 75 bp single end reads. (4) An X-ray mutagenised *mnd* mutant in barley cv. Saale was out-crossed to cv. Barke to generate a mapping population [11]. Exome capture was performed on two pools of DNA from 18 mutant and 30 wild type plants, respectively. Sequencing was carried out on Illumina Hiseq2000 as paired reads (2X 100 bp).

### Simulations

For mapping by sequencing to be successful, we need ordered reference sequence to place variants from bulk segregant sequence data on chromosomes to identify the region of genome with linkage disequilibrium. We wanted to test the impact of fragmented nature of *de-novo* genome assemblies in identifying the genomic region with linkage disequilibrium. To avoid variability resulting from parameters of genome assembly, variant calling and sequencing depth we have used variant data from BSS experiments that had been previously published to identify causative mutation using Arabidopsis ordered reference genome. Arabidopsis BSS data for *hasty* [12] (a backcross) and *sup2* [9] (an outcross) were used for variant calling against Arabidopsis Col-0 TAIR10 genome. Sequencing reads from pooled samples of mutant bulks and parents were quality filtered and trimmed using Trimmomatic v0.33 [13] (with options: ILLUMINACLIP:Trimmomatic provided Illumina adapter file:2:30:10 HEADCROP:10 LEADING:10 TRAILING:10 SLIDINGWINDOW:4:15 MINLEN:31) aligned using BWA [14] mem (v0.7.12) with default settings and bamfiles were generated using samtools [15] (v1.0). Mpileup file generated using samtools (-q 20 -Q 15 -d 20xmean_depth) and variant calls were generated using varScan [16] (v2.3.9, options: mpileup2cns –variants 1 –output-vcf 1 –strand-filter 1). Homozygous variant calls from background bulk data were subtracted from mutant bulk data. Remaining mutant bulk data variants from backcross and outcross datasets were used in respective simulations. We have used paired end reads of miR159a parent [12] to assemble Arabidopsis genome. Reads were quality filtered and trimmed using Trimmomatic v0.33. Genome assembly was done using SOAPdenovo [17] v2.40 with Multi-Kmer method (SOAPdenovo-127mer all -K 25 -d 1 -R -M 1 -m 95 -E -F). Assembled scaffolds of ≥300bp were selected resulting in assembly size of 117.6 Mb (*n* =18,267), with a N50 length of 20.3 Kb. Resulting assembly scaffold lengths were modelled against normal, log-normal and exponential distribution; and found to follow a log-normal distribution (Additional file 1: Figure S1). Arabidopsis genome was randomly fragmented using log-normal distribution (mean: 7.88 and alpha: 1.56) to generate a 1000 fragmented genome assemblies each for outcross and backcross experiment. Position and chromosomal order of individual fragments in each generated assembly is known. Background subtracted variant data from the mutant bulk were assigned to respective fragments to generate 1000 assemblies with variant data from bulk segregant sequencing.

### CHERIPIC analysis

***Arabidopsis***: De-novo assembly made for bulk segregant simulation analysis was used as a reference to call variants using single end whole genome reads of outcrossed bulked individuals showing *sup2* [9] phenotype, and two parents Col-T and Ws. Variant analysis was carried out as mentioned in the “Simulations” section. Assembly and variant files are provided as inputs to CHERIPIC. CHERIPIC takes multiple inputs for background variants, as outcrosses involve two polymorphic parents and as was the case for *sup2* experiment. Removing background variants from both parents would help in removing candidates not linked to the phenotype but arising from regions with suppressed recombination.

***Maize***: Single end RNA-seq reads from combined bulks of both *gl3* mutant and non-mutant phenotype [10] were quality filtered and trimmed using Trimmomatic v0.33 (options: ILLUMINACLIP:ilmn_adapters.fa:2:30:10 HEADCROP:13 LEADING:10 TRAILING:10 SLIDINGWINDOW:4:15 MINLEN:25). Assembly was carried out using Trinity [18] v2.0.6 (options: –seqType fq –max_memory 50G –CPU 64 –full_cleanup) by using sequences from both bulks. Assembly using default parameters resulted in 33,563 transcript sequences, which were clustered using CD-HIT [19] with identity threshold of -c 0.975, resulting in 29,288 transcripts.

***Barley***: Wildtype pool of paired end exome sequence data from *mnd* bulk segregant sequences [11] were quality filtered and trimmed using Trimmomatic v0.33 and were assembled using SOAPdenovo v2.40 with Multi-Kmer method (SOAPdenovo-127mer all -K 25 -d 1 -R -M 1 -m 95 -E -F). Assembled scaffolds of ≥300bp has resulted in sequence of 166.5 Mb (n=253137) and a N50 length of 0.72 Kb.

## Availability and requirements

**Project name:** CHERIPIC **Project home page:**https://github.com/TeamMacLean/cheripic**Operating system(s):** Linux and Mac OS **Programming language:** Ruby **Other requirements:** CHERIPIC has light computational requirements. It will run on Linux or Mac OS operating system with 2GHz CPU and minimally 8GB RAM. Higher RAM may be required if input files are large. **License:** MIT **Any restrictions to use by non-academics:** None

## Additional file

Additional file 1 Supplemental Figures. **Figure S1.** Frequency distribution of assembly fragment lengths follow a log-normal distribution. **Figure S2.** Outline of CHERIPIC method. **Figure S3.** Variants selected by CHERIPIC for Arabidopsis *sup2* data were presented on all five chromosomes. **Figure S4.** All variants selected by CHERIPIC for maize *gl3* data were presented on all ten chromosomes. **Figure S5.** Variants selected by CHERIPIC for barley *mnd* data were presented on all seven chromosomes. (PDF 854 kb)

## Abbreviations

BSS: Bulk segregant sequencing
CHERIPIC: Computing homozygosity enriched regions in genomes to prioritise identification of candidate variants
HMES: Homozygosity enrichment score
MBC: Map based cloning
MBS: Mapping by sequencing
