# Supplementary material for: Rapid fine mapping of causative mutations from sets of unordered, contig-sized fragments of genome sequence
Source: BMC Bioinformatics. 2019 Jan 7;20:9. doi: 10.1186/s12859-018-2515-5 (PMC6323790; doi:10.1186/s12859-018-2515-5)
Supplement: Supplementary file 1 — Supplemental Figures. Figure S1. Frequency distribution of assembly fragment lengths follow a log-normal distribution. Figure S2. Outline of CHERIPIC method. Figure S3. Variants selected by CHERIPIC for Arabidopsis sup2 data were presented on all five chromosomes. Figure S4. All variants selected by CHERIPIC for maize gl3 data were presented on all ten chromosomes. Figure S5. Variants selected by CHERIPIC for barley mnd data were presented on all seven chromosomes. (PDF 854 kb) [file 12859_2018_2515_MOESM1_ESM.pdf]

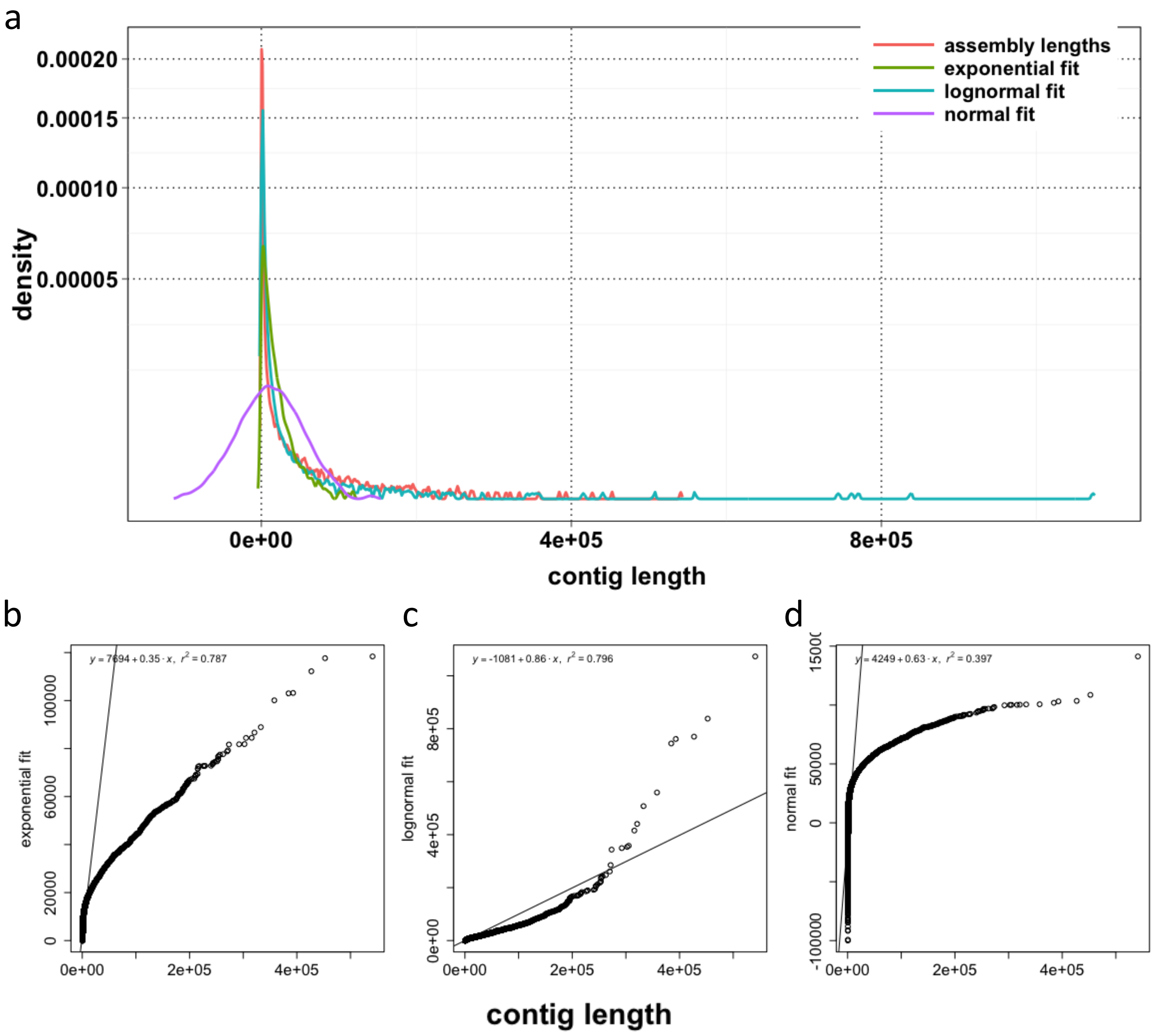

Figure S1. Frequency distribution of assembly fragment lengths follow a log-normal distribution. (a) Distribution of genome assembly fragment lengths of Arabidopsis and fitted exponential, log-normal and normal distributions. (b, c, d) Q-Q plots fitting observed and estimated lengths for (b) exponential, (c) log-normal and (d) normal distribution.

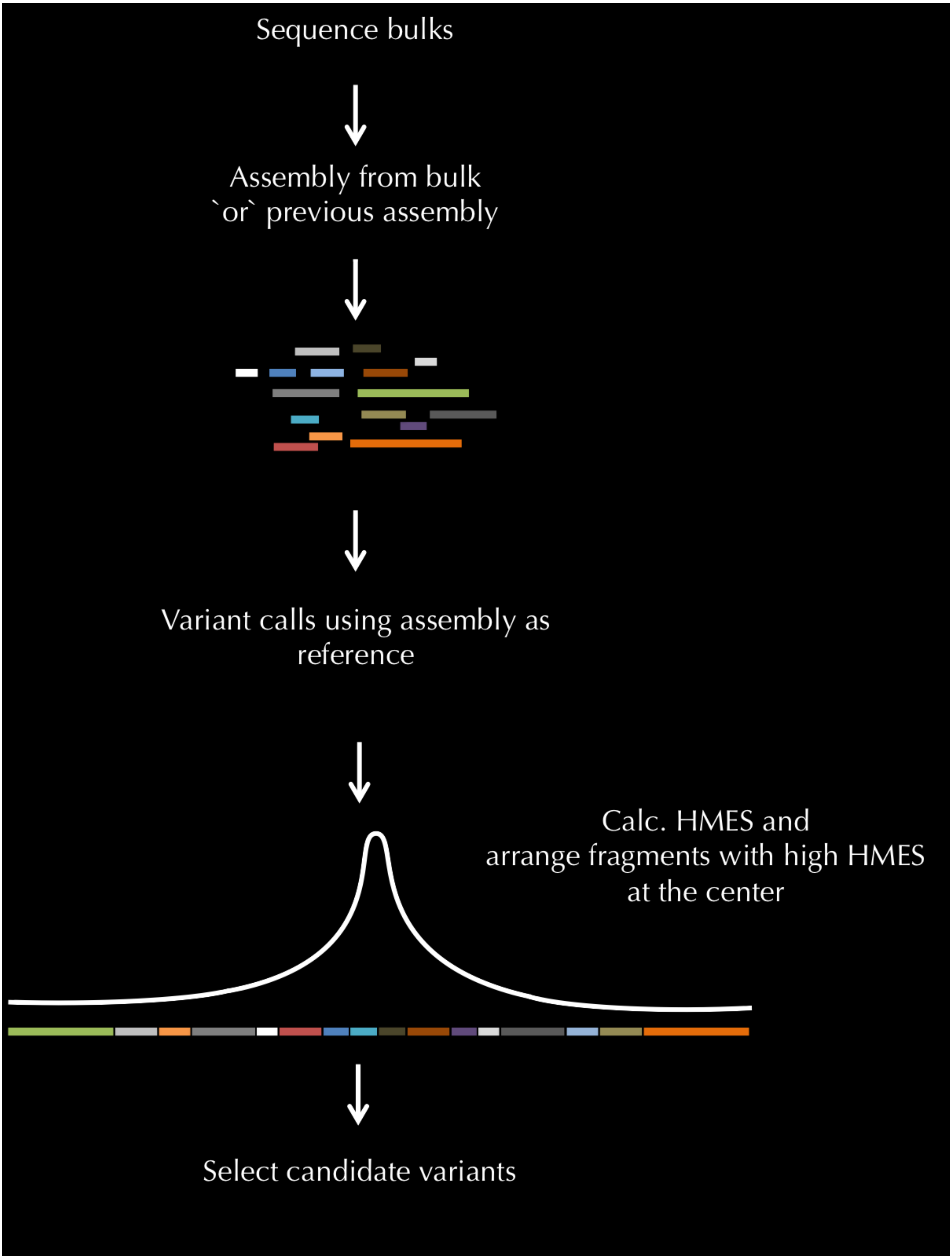

Figure S2. Outline of CHERIPIC method

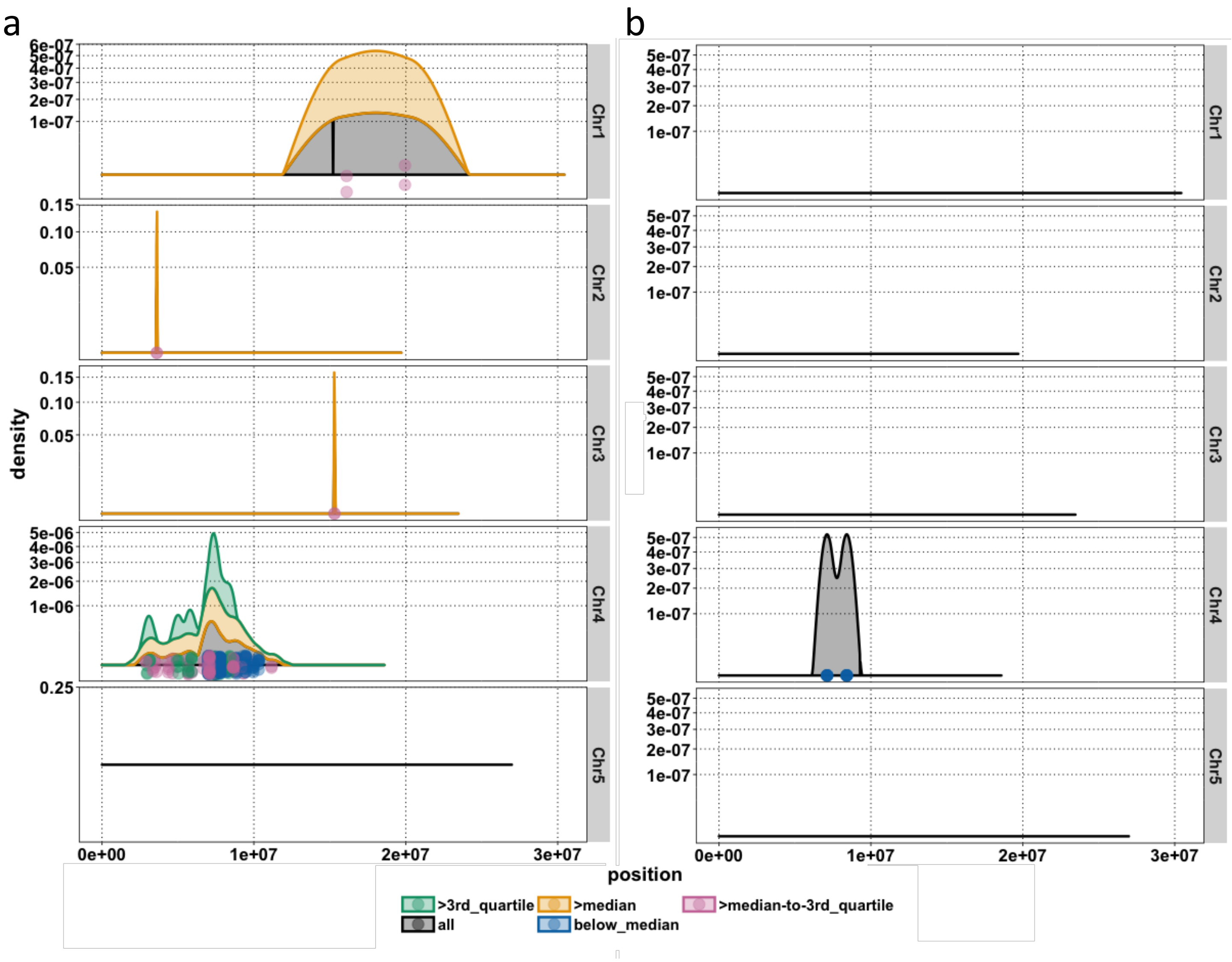

Figure S3. Variants selected by CHERIPIC for Arabidopsis *sup2* data were presented on all five chromosomes. (a) All variants were depicted; (b) Top five percentile *HMES* variants were depicted.

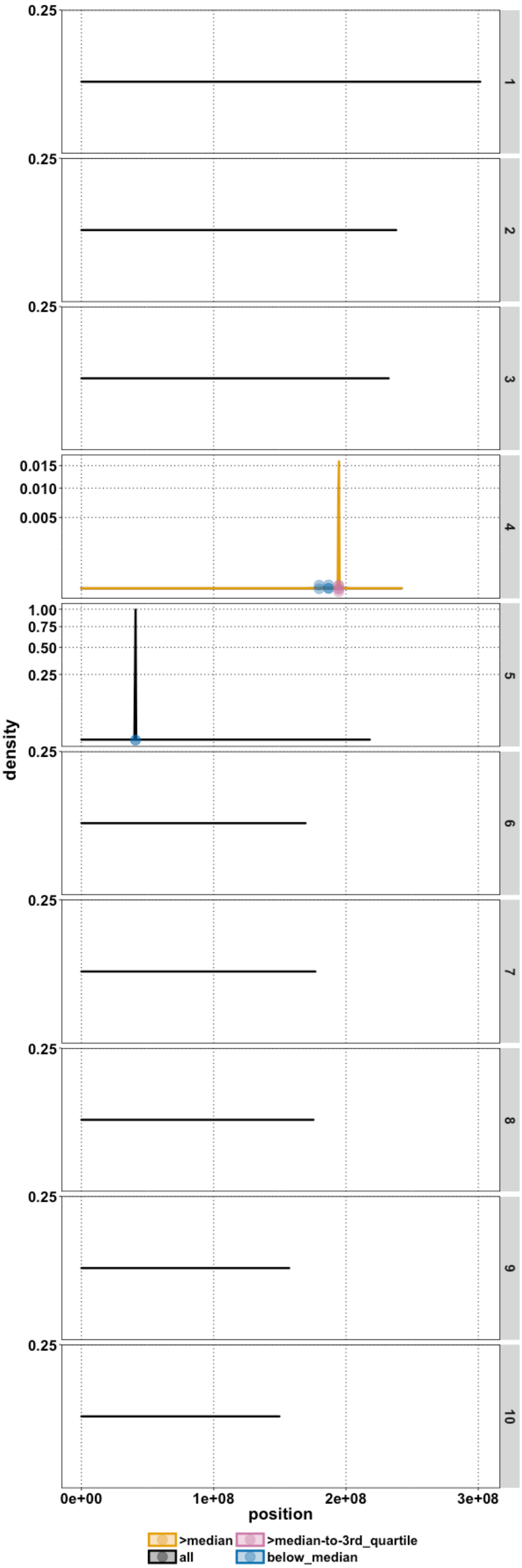

Figure S4. All variants selected by CHERIPIC for maize *gl3* data were presented on all ten chromosomes.

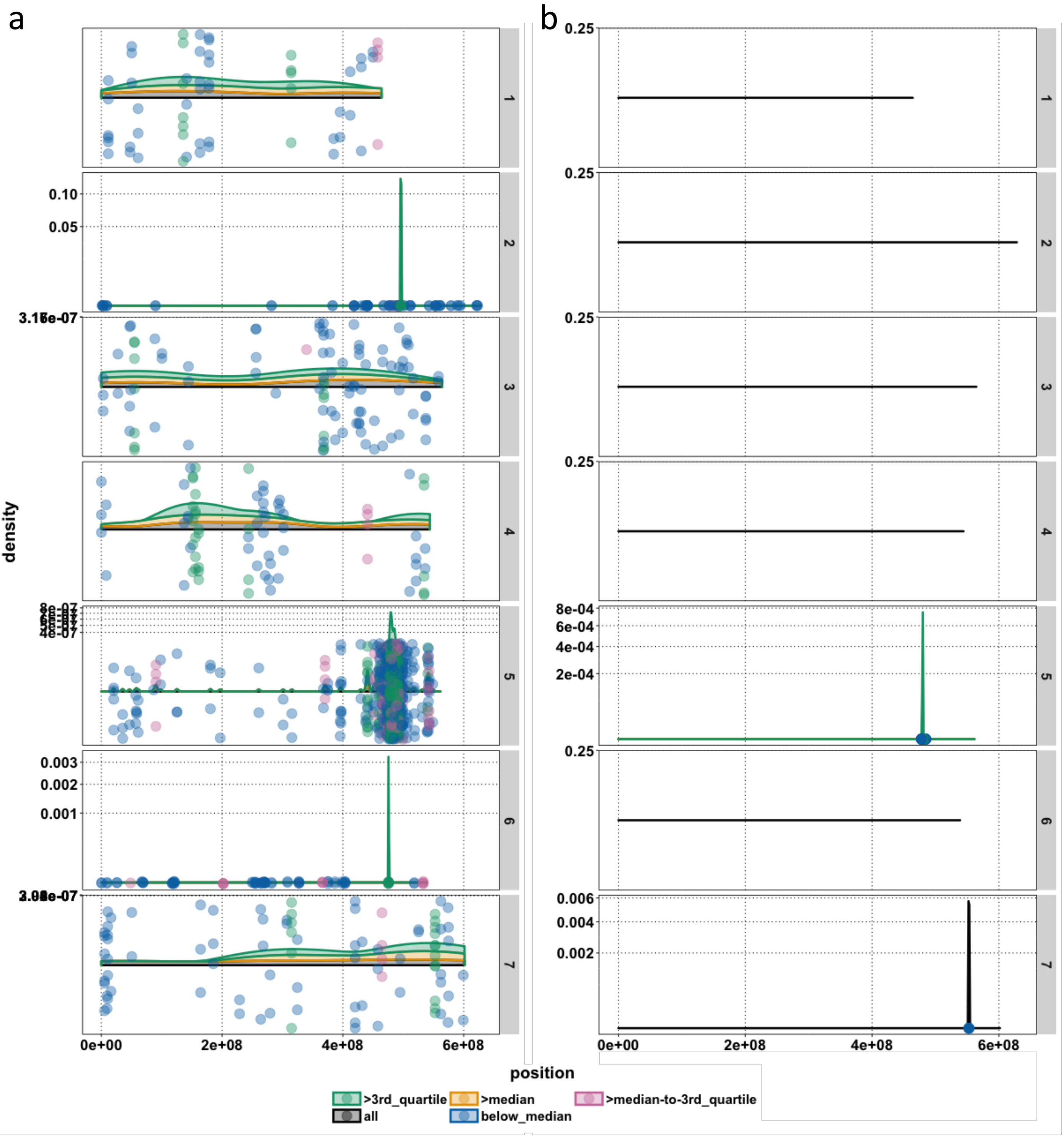

Figure S5. Variants selected by CHERIPIC for barley *mnd* data were presented on all seven chromosomes. (a) All variants were depicted; (b) Top five percentile *HMES* variants were depicted.
